# Astringents in Chronic Conjunctivitis

**Published:** 1880-05

**Authors:** F. M. Wilson

**Affiliations:** Bridgeport, Conn.


ASTRINGENTS IN CHRONIC CONJUNCTIVITIS, BY DR. F. M. WILSON, BRIDGEPORT, CONN.

THE treatment of a chronically inflamed conjunctiva, is often tedious both to physician and patient. A prominent part of this treatment usually consists in the use of some one of the many local applications. With the design of ascertaining what applications were most preferred, circulars were sent to members of the State Medical Society and of the American Ophthalmological Society, and to about half a dozen medical friends not members of either. One hundred and two opinions have been received from general practitioners and forty-one from specialists.

Twenty-two different drugs were mentioned. The following tables contain the more important ones, and the numbers who claim for each first rank :

*1. General Practitioners.*

| | |
|---|---|
| Argenti Nitras, . . . . . | 38. |
| Zinci Sulphas, . . . . | 32. |
| Cupri Sulphas, . . . . . | 15. |
| Plumbi Acetas, . . . . | 5. |
| Alumen, . . . . . | 3. |
| Other drugs, . . . . | 9. |
| | 102. |

*2. Specialists.*

| | |
|---|---|
| Argenti Nitras, . . . . . | 18. |
| Cupri Sulphas, . . . . | 10. |
| Alumen, . . . . . | 4. |
| Zinci Sulphas, . . . . | 2. |
| Other drugs, . . . . . | 4. |
| Uncertain, . . . . . | 3. |
| | 41. |

As will be readily seen, there is a preference for argenti nitras, both on the part of general practitioners and also of specialists. Table 2d is especially significant, representing as it does the treatment of many thousands of cases. The most noticeable difference between the two is the greater number of general practitioners who, with Drs. Roosa, of New York, and Strawbridge, of Philadelphia, prefer zinci sulphas.

The inquiry also brought out an important difference in mode of application.

A majority of general practitioners depend chiefly upon collyria, which they rely upon the patient to drop into the eye.

Specialists, as a rule, place their chief dependence upon stronger applications, which they never trust out of their own hands.

